# Supplementary material for: Structure of Machupo virus polymerase in complex with matrix protein Z
Source: Nat Commun. 2021 Oct 25;12:6163. doi: 10.1038/s41467-021-26432-3 (PMC8546121; doi:10.1038/s41467-021-26432-3)
Supplement: Supplementary file 1 — Supplementary Information [file 41467_2021_26432_MOESM1_ESM.pdf]

Supplementary information

Structure of Machupo virus polymerase in complex with matrix protein Z

Jun Ma[1,4], Shuangyue Zhang[1,2,4], Xinzheng Zhang[1,2,3]

[1] National Laboratory of Biomacromolecules, CAS Center for Excellence in Biomacromolecules, Institute of Biophysics, Chinese Academy of Sciences, Beijing, 100101, China

[2] University of Chinese Academy of Sciences, Beijing, 100049, China

[3] Center for Biological Imaging, CAS Center for Excellence in Biomacromolecules, Institute of Biophysics, Chinese Academy of Sciences, Beijing, 100101, China

[4] These authors contributed equally: Jun Ma, Shuangyue Zhang

Correspondence: Xinzheng Zhang (xzzhang@ibp.ac.cn)

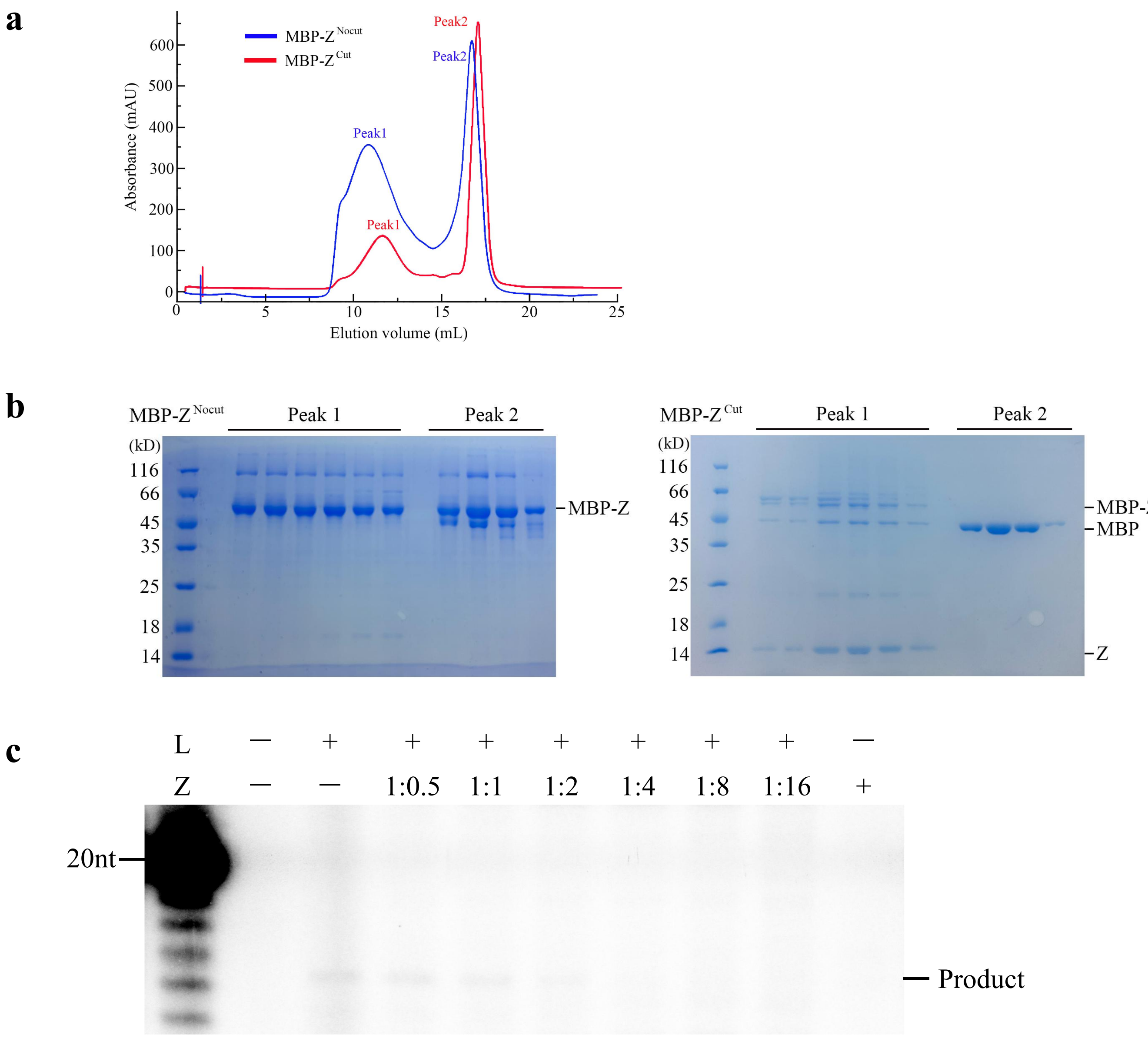

Supplementary Figure 1. Biochemical Characterization of MACV Z protein. a Gel filtration analysis of MBP-Z fusion protein with Superdex 200 Increase 10/300 GL. Blue, MBP-Z fusion protein after Ni column; Red, monomeric MBP-Z fusion protein digested by PPase. **b** The peaks of gel filtration profile are confirmed by subsequent coomassie-stained SDS-PAGE. Molecular weights (in kilodaltons) of marker are shown on the left, and bands are labeled on the right. **c** In vitro polymerase activity assay for MACV L protein inhibited by MBP-Z fusion protein. The different mole ratio between the L and Z proteins is indicated. The inhibitory effect increases with an increasing amount of MBP-Z fusion protein. The data shown above are representative results of more than two independent experiments.

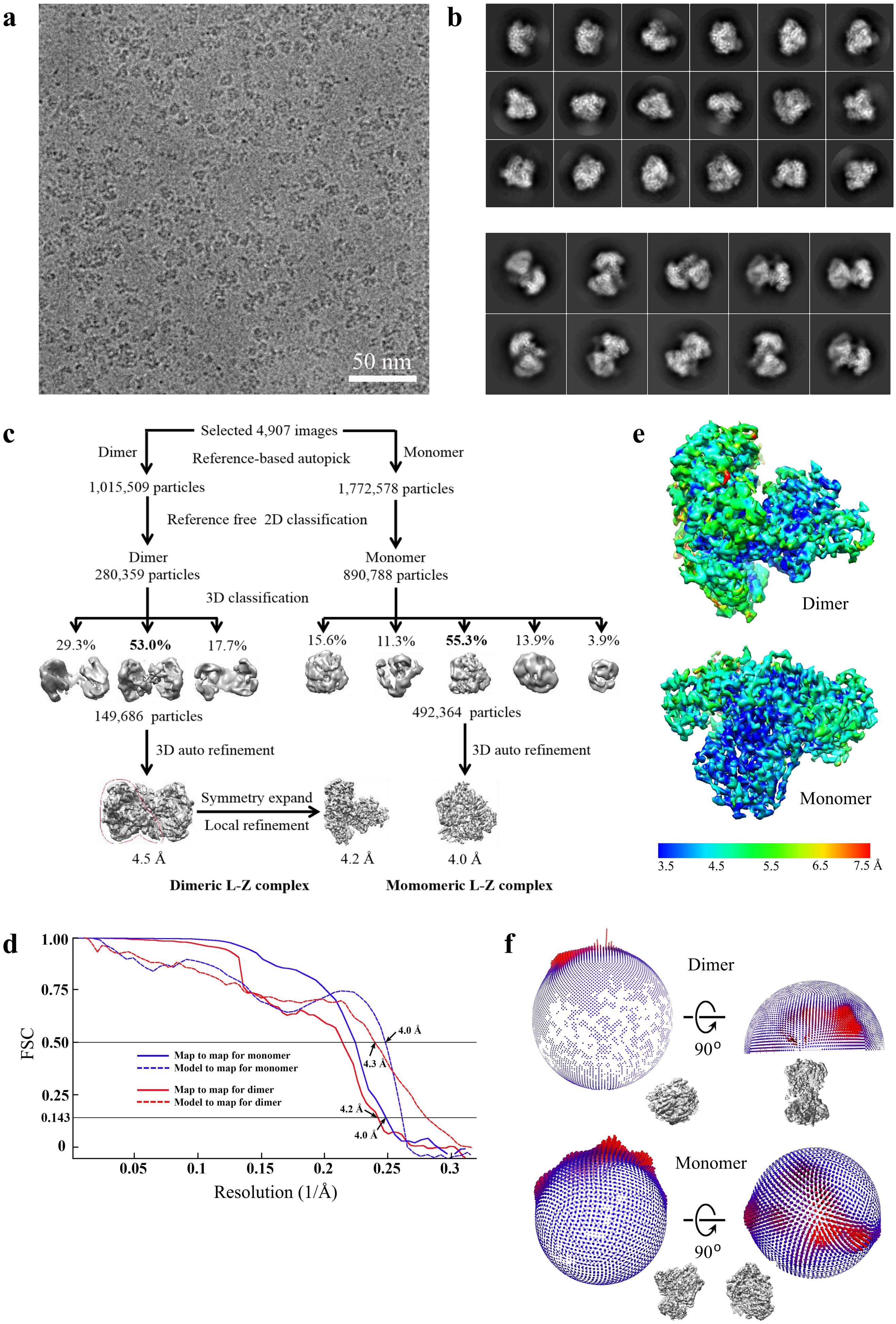
a
50 nm
b
c
Selected 4,907 images
Dimer
Reference-based autopick
Monomer
1,015,509 particles
1,772,578 particles
Reference free 2D classification
Dimer
280,359 particles
Monomer
890,788 particles
3D classification
29.3%
53.0%
17.7%
15.6%
11.3%
55.3%
13.9%
3.9%
149,686 particles
492,364 particles
3D auto refinement
3D auto refinement
Symmetry expand
Local refinement
4.5 Å
4.2 Å
4.0 Å
Dimeric L-Z complex
Momomeric L-Z complex
e
Dimer
Monomer
3.5
4.5
5.5
6.5
7.5 Å
d
FSC
1.00
0.75
0.50
0.25
0.143
0
Map to map for monomer
Model to map for monomer
Map to map for dimer
Model to map for dimer
4.0 Å
4.3 Å
4.2 Å
4.0 Å
0.05
0.1
0.15
0.2
0.25
0.3
Resolution (1/Å)
f
Dimer
90°
Monomer
90°

Supplementary Figure 2. Single-particle cryo-EM analysis of MACV L-Z complex. **a** Representative cryo-EM image of MACV L-Z complex (one out of 4,907 micrographs). **b** Representative reference-free 2D-class averages of monomeric (upper) and dimeric complex (lower). **c** Data-processing workflow for MACV L-Z complex. **d** The gold standard Fourier shell correlation (FSC) curves for the reconstruction. FSC curves between two half maps (monomer: blue line, dimer: red line) are indicated with resolutions at FSC = 0.143. FSC curves between model and map (monomer: blue dashed line, dimer: red dashed line) are indicated with resolutions at FSC = 0.5. **e** Local resolution maps for dimeric (upper) and monomeric (lower) complex estimated by RasMap. **f** Angular distribution of dimeric (upper) and monomeric (lower) particle.

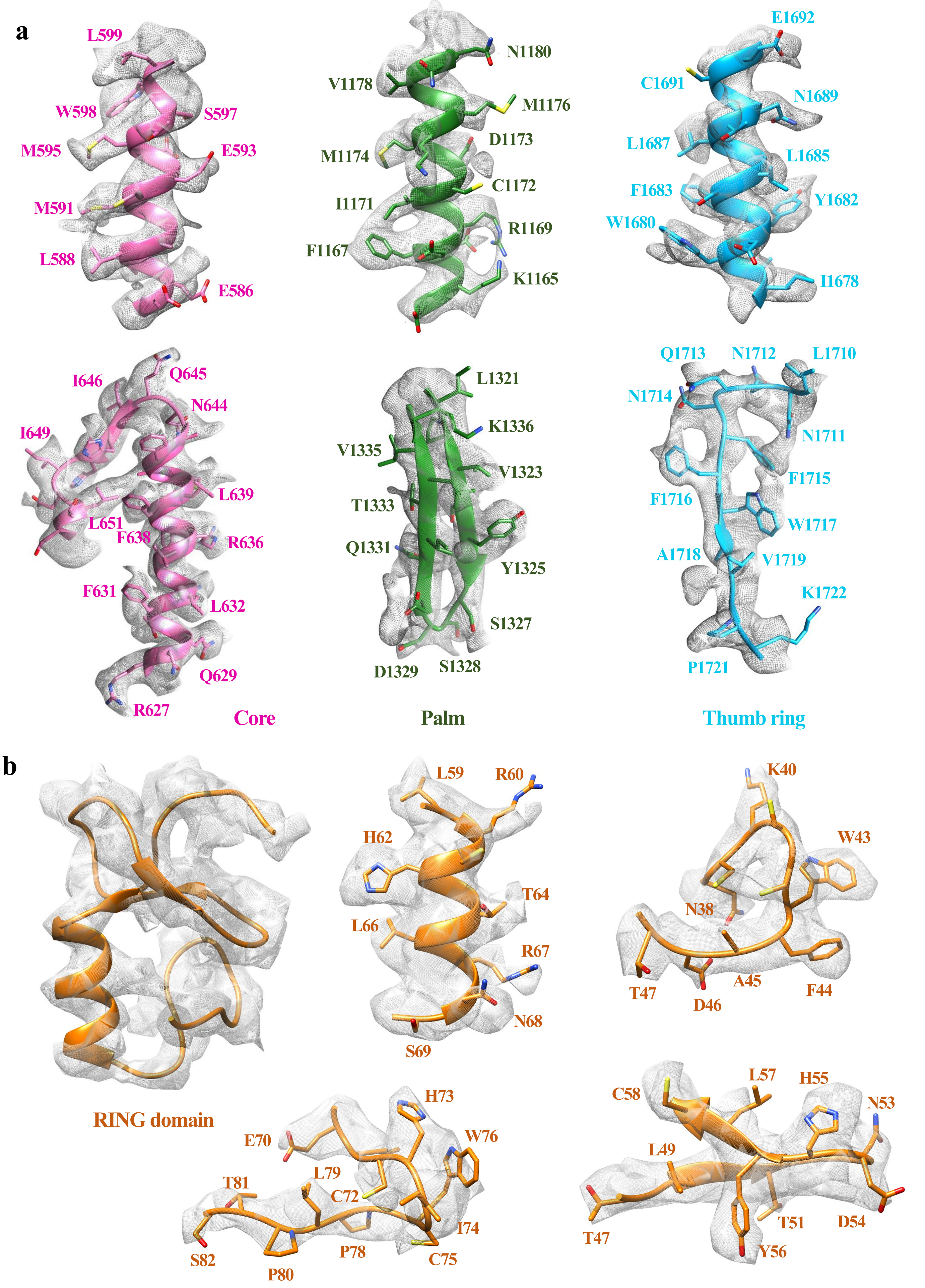

Supplementary Figure 3. Representative densities and atomic models of monomeric MACV L-Z complex. a Representative densities and atomic models of selected secondary structures of L. **b** Representative densities and atomic models of selected secondary structures of Z. The densities are shown as gray meshes. The corresponding models are shown as ribbon colored as Figure 1a, and selected side chain are shown as sticks and labeled.

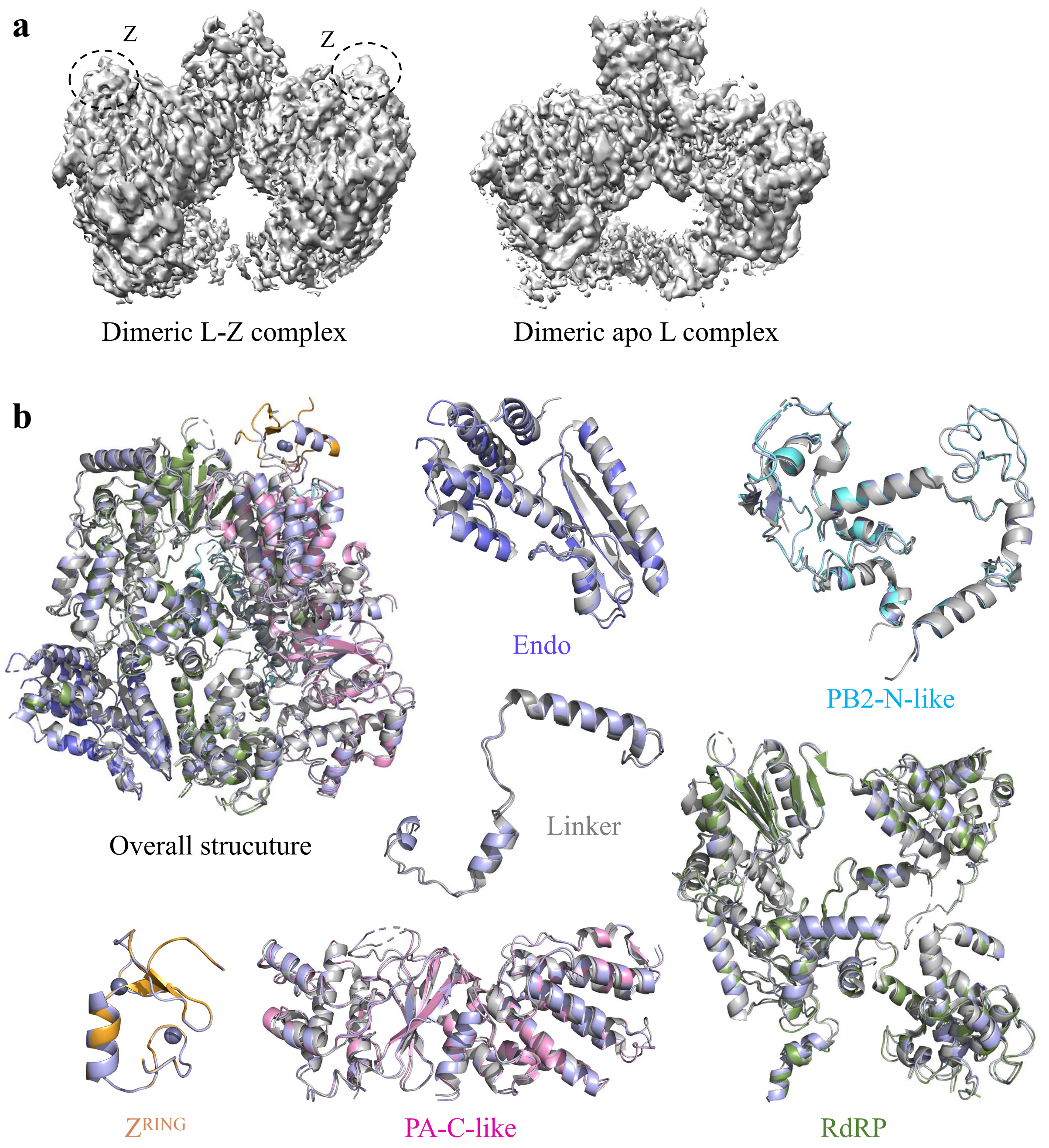

Supplementary Figure 4. Structural comparison of MACV L protein in different states. a Comparison of density maps between dimeric L-Z complex and apo L. The densities of the Z protein in the dimeric L-Z complex are indicated by black dashed circles. **b** Superposition of monomeric L-Z complex with monomer in dimeric L-Z complex and apo L protein. The overall structure and domains are superposed, respectively. Domains of monomeric L-Z complex are colored as in Figure 1a, while monomer in dimeric L-Z complex is colored blue white and apo L protein colored gray.

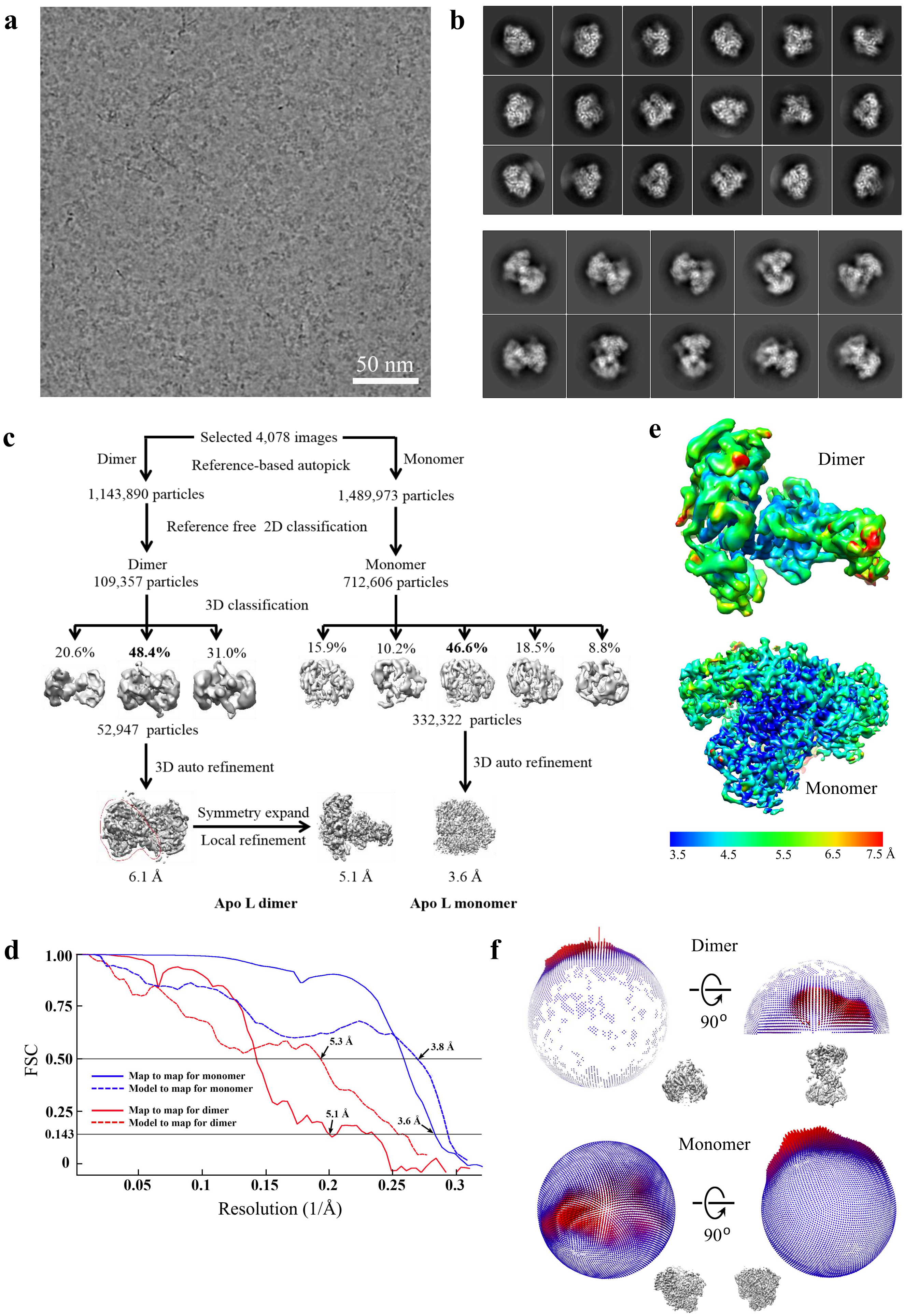

a
50 nm
b
c
Selected 4,078 images
Dimer
Reference-based autopick
Monomer
1,143,890 particles
1,489,973 particles
Reference free 2D classification
Dimer
109,357 particles
Monomer
712,606 particles
3D classification
20.6%
48.4%
31.0%
15.9%
10.2%
46.6%
18.5%
8.8%
52,947 particles
332,322 particles
3D auto refinement
3D auto refinement
Symmetry expand
Local refinement
6.1 Å
5.1 Å
3.6 Å
Apo L dimer
Apo L monomer
d
FSC
1.00
0.75
0.50
0.25
0.143
0
Map to map for monomer
Model to map for monomer
Map to map for dimer
Model to map for dimer
5.3 Å
3.8 Å
5.1 Å
3.6 Å
0.05
0.1
0.15
0.2
0.25
0.3
Resolution (1/Å)
e
Dimer
Monomer
3.5
4.5
5.5
6.5
7.5 Å
f
Dimer
90°
Monomer
90°

Supplementary Figure 5. Single-particle cryo-EM analysis of apo MACV L protein. **a** Representative cryo-EM image of apo MACV L protein (one out of 4,078 micrographs). **b** Representative reference-free 2D-class averages of monomer (upper) and dimer (lower). **c** Data-processing workflow for apo MACV L protein. **d** The gold standard Fourier shell correlation (FSC) curves for the reconstruction. Curves are colored and resolutions indicated as Supplementary Figure 2d. **e** Local resolution maps for dimer (upper) and monomer (lower). **f** Angular distribution of dimeric (upper) and monomeric (lower) particles included in the final 3D reconstructions, with the cryo-EM maps shown in gray.

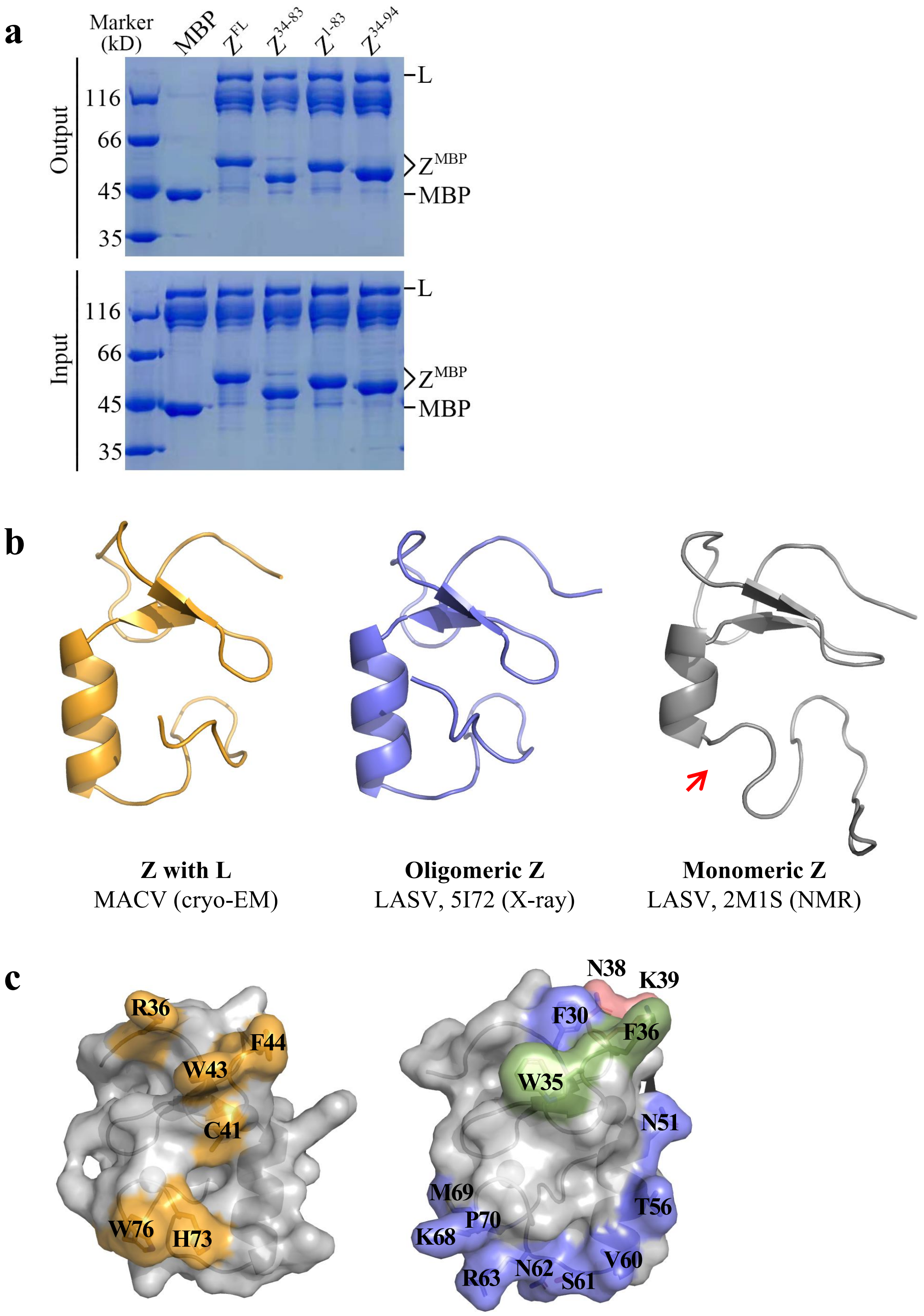

Supplementary Figure 6. Structural comparison of MACV and LASV Z proteins. a In vitro pull-down assay of different truncated Z proteins with MACV L protein. Experiments were repeated independently for more than two times. **b** Structural comparison of MACV Z protein in L-Z complex, LASV Z protein in oligomer (PDB 5I72, by crystallography) and monomer form (PDB code 2MIS, by NMR), colored orange, slate and gray, respectively. The short α-helix is indicated by a red arrow. **c** Comparison of residues of MACV and LASV Z proteins involved in the interactions. Left, MACV Z protein is represented as gray transparent surface, with residues in interaction with L protein colored orange. Right, residues of LASV Z protein involved in polymerization is colored blue and smudge, with residues involved in interaction with eIF4e colored pink and smudge.

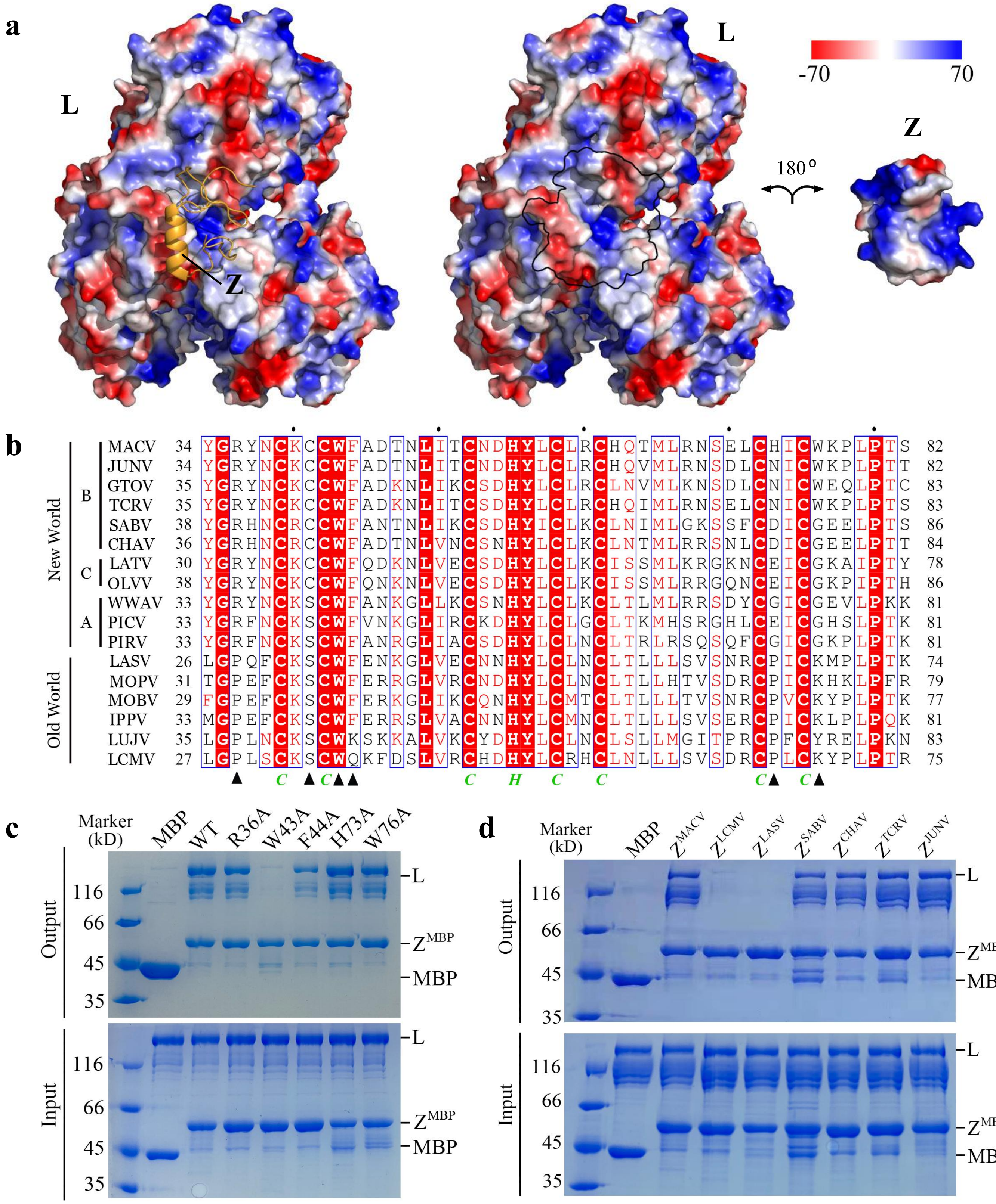

Supplementary Figure 7. The interaction between the L and Z proteins. a The electrostatic surfaces of the L and Z proteins are shown in an open-book representation. The electrostatic surface is colored blue for positive charges and red for negative charges. The binding interface on L of the Z protein is outlined with a black solid line. **b** Multiple-sequence alignment for RING domain of the Z protein from different arenaviruses. The sequences are classified as Old World and New World arenaviruses marked left. Residues involved in the interaction with L are marked below with black solid triangle, while conserved cysteines and histidine involved in Zn binding are marked below with green italicized C or H. Uniprot accession codes of Z: MACV, Q6IUF9; JUNV, Q6IVU5; GTOV, Q6UY71; TCRV, Q88470, SABV, Q6UY62, CHAV, B2C4J2; LATV, A9JR44; OLVV, B0BLK7; WWAV, B2ZDY1; PICV, Q915A4; PIRV, Q6RSS3; LASV, O73557; MOPV, G3LUW9; MOBV, Q27YE6; IPPV, Q27YE2; LCMV, P18541; LUJV, C5ILC3. **c** In vitro pull-down assay of MACV MBP-Z fusion protein mutants involving in the L-Z interaction with the MACV L. **d** In vitro pull-down assay of MBP-Z fusion proteins from different arenaviruses with the MACV L. Experiments in (**c**) and (**d**) were repeated independently for more than two times.

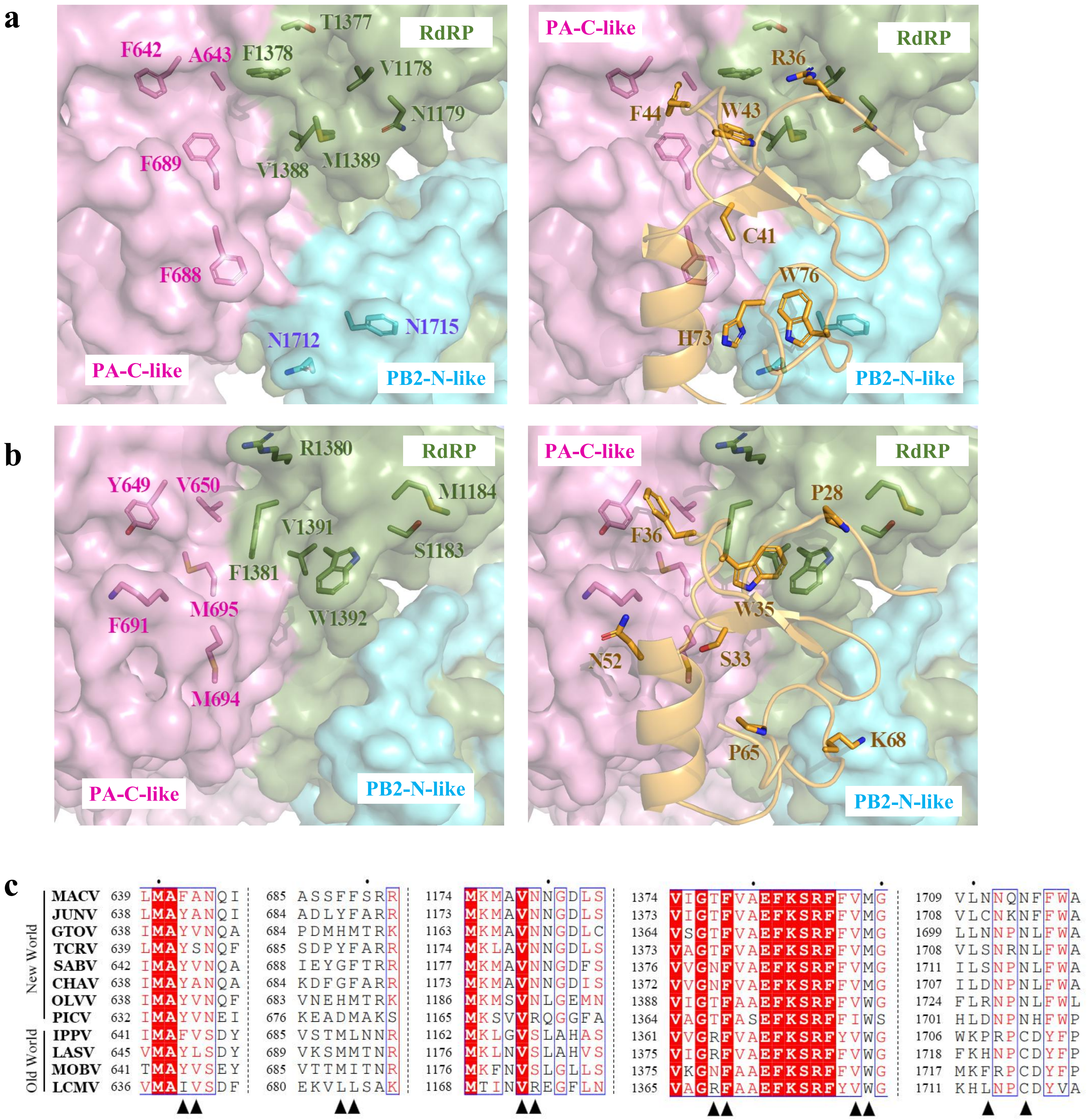

Supplementary Figure 8. Comparison of binding sites of the L-Z complex between MACV and LASV. **a**, **b** Binding sites of the L-Z complex. MACV (**a**) and LASV (**b**) L protein (left) are represented as transparent surface. The domains are indicated and colored as in Figure 1a with the side chain of key residues shown as sticks and labeled. MACV and LASV Z protein (right) are in orange ribbons representation with key residues shown as sticks and labeled. The model of the LASV L-Z complex was produced by superposition the cryo-EM structure of LASV L(PDB 6KLC) and the crystal structure of LASV Z (PDB 5I72) with the MASV L-Z complex. **c** Multiple-sequence alignment for L of the regions involved in the interaction with Z. The sequences are classified as Old World and New World arenaviruses marked left. Residues involved in the interaction with Z are marked below with black solid triangle. Uniprot accession codes of L: MACV, Q6IUF8; JUNV, Q6XQI4; GTOV, Q6UY70; TCRV, P20430; SABV, Q6UY61; CHAV, B2C4J3; OLVV, Q6XQH7; PIRV, Q6RSS2; IPPV, Q27YE1; LASV, O09705; MOBV, Q27YE5; LCMV, P14240.

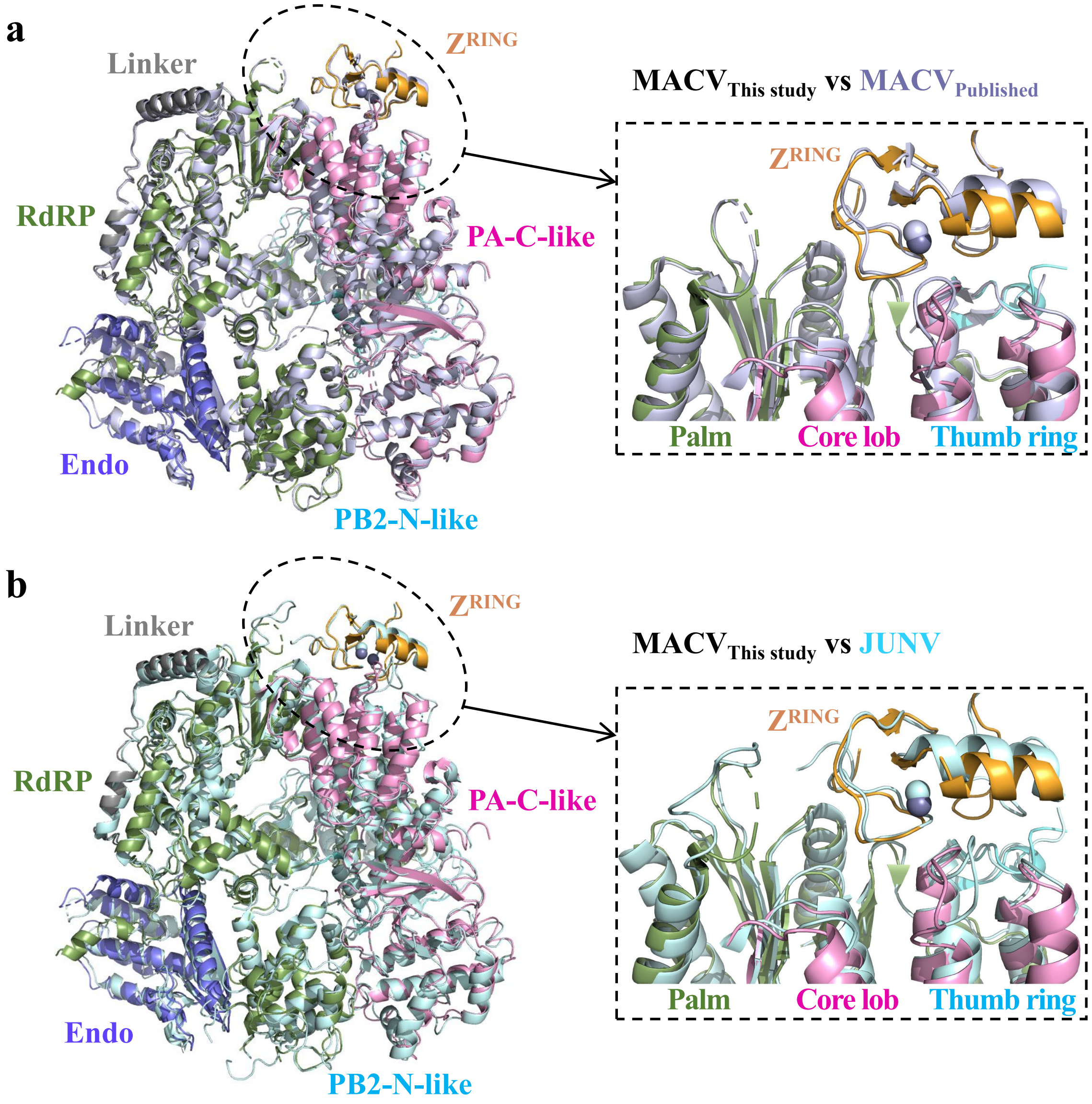

Supplementary Figure 9. Structural comparison of the MACV L-Z complex in this study with the MACV and JUNV L-Z complexes published. a, **b** Left, superposition of the overall structure of the MACV L-Z complex in this study with the MACV L-Z complex (PDB 7CKM) (**a**) and the JUNV L-Z complex (PDB 7EJU) (**b**) published. Right, magnified view of the L-Z interfaces indicated by black dashed circles on the left. Domains of the MACV L-Z complex in this study are indicated and colored as in Figure 1a, while the MACV L-Z complex published is colored blue white and the JUNV L-Z complex colored pale cyan.

	MACV L-Z complex		apo MACV L	
Monomer	EMD-31975, 7VGQ		EMD-31985, 7VH3	
Dimer	EMD-31983, 7VH1		EMD-31984, 7VH2	
**Data collection and processing**				
Microscope	FEI Arctica		FEI Titan Krios	
Detector	Gatan K2 Summit		Gatan K2 Summit	
Magnification (nominal/calibrated)	130,000		105,000	
Voltage (kV)	200		300	
Electron exposure (e$^-$/Å^2)	60		60	
Defocus rang (µm)	1.0-3.0		0.8-3.0	
Pixel size (Å)	0.8		0.82	
	**Monomer**	**Dimer**	**Monomer**	**Dimer**
Symmetry imposed	C1	C2	C1	C2
Initial particle images (no.)	1,772,578	1,015,509	1,489,973	1,143,890
Final particle images (no.)	492,364	149,686	332,322	52,947
Map resolution (Å)	4.0	4.2	3.6	5.1
FSC threshold	0.143	0143	0.143	0143
Map resolution range (Å)	3.5-7.5	3.5-7.5	3.5-7.5	3.5-7.5
**Model refinement and validation**				
Initial model used (PDB code)	6KLD	6KLH	6KLD	6KLH
Model resolution (Å)	4.0	4.2	3.6	5.1
Model resolution range (Å)	∞-4.0	∞-4.2	∞-3.6	∞-5.1
Map sharpening *B* factor (Å^2)	-230	-193	-180	-204
Model composition				
Non-hydrogen atoms	13156	13156	12749	12749
Protein residues	1661	1661	1612	1662
Ligands	2	2	-	-
B factors (Å^2)				
Protein	6.85	89.44	27.01	208.39
Ligand	21.89	115.64	-	-
R.m.s. deviations				
Bond lengths (Å)	0.010	0.010	0.006	0.008
Bond angles (°)	1.49	1.51	1.33	1.45
Validation				
MolProbity score	2.04	1.93	1.96	2.16
Clashscore	7.14	5.96	8.07	9.95
Rotamer outliers (%)	0.81	0.74	0.90	0.76
Ramachandran plot				
Favored (%)	85.94	87.85	91.02	86.16
Allowed (%)	13.87	11.91	8.53	13.72
Disallowed (%)	0.18	0.25	0.44	0.13

Supplementary Table 1. Cryo-EM data collection and validation statistics.

a The codon-optimized DNA sequence of MACV L gene

```
ATGGACGAGTACGTGCAGGAACTGAAGGGCCTGATCCGTAAGCACATCCCTGACCGCTGCGAGTTCGCTCACCAGAAG
GTCACTTTCCTGTCCCAGGTCCACCCTTCCCCCCTGCTGACTGAAGGCTTCAAGCTGCTGAGCTCCCTGGTGGAGCTG
GAAAGCTGCGAGGCCCACGCCTGCCAGGCCAACACCGACCAGCGCTTCGTGGACGTCATCCTGTCCGACAACGGTATC
CTGTGCCCTACTCTGCCTAAGGTCATTCCCGACGGTTTCAAGCTGACCGGCAAGACCCTGATCCTGCTGGAGACTTTC
GTGCGTGTGAACCCCGACGAATTTGAGAAGAAGTGGAAGGCCGACATGTCCAAGCTGCTGAACCTGAAGCACGACCTG
CAGAAGAGCGGCGTCACCCTGGTCCCCATCGTGGACGGCCGTAGCAACTACAACAACCGTTTCGTCGCCGACTGGGTC
ATCGAACGCATGCGCTGGCTGCTGATCGAAATCCTGAAGGCTTCCAAGTCCATGCTGGAAATCGACATCGAGGACCAG
GAGTACCAGCGCCTGATCCACTCCCTGTCCAACGTGAAGAACCAGAGCCTGGGCCTGGAGAACCTGGAGCACCTGAAG
CGCAACTCCCTGGACTACGACGAACGTCTGAACGAAAGCCTGTTCATCGGCCTGAAGGGCGACATCCGCGAATCCACT
GTCCGTGAGGAGCTGATCAAGCTGAAGATGTGGTTCAAGGACGAAGTGTTCTCCAAGGGCCTGGGCAAGTTCAAGCTG
ACTGACCGCCGTGAGCTGCTGGAGTCCCTGTCCTCCCTGGGCGCTCACCTGGACTCCGACGTCTCCTCCTGCCCCTTC
TGCAACAACAAGCTGATGGAGATCGTCTACAACGTGACCTTCTCCAGCGTGGAGCGCACTGACGGTGCTGCCACCGTC
GACCAGCAGTTCTCCACCACTCACACTAACATCGAGAAGCACTACCTGTCCGTCCTGAGCCTGTGCAACAAGATCAAG
GGCCTGAAGGTCTTCAACACCCGCCGCAACACCCTGCTGTTCCTGGACCTGATCATGGTCAACCTGATGGTCGACATC
TCCGAGTCCTGCCAGGACGCTATCGAGTCCCTGCGTAAGTCCGGTCTGATCGTCGGTCAGATGGTCATGCTGGTGAAC
GACCGCGTGCTGGACATCCTGGAGGCTATCAAGCTGATCCGTAAAAAGATCGGTACTAACCCCAACTGGGTCAAGAAC
TGCTCCAAGATCCTGGAGCGCAGCCACCCTGAGATCTGGCTGCAGCTGAACACCCTGATCCGCCAGCCTGACTTCAAC
AGCCTGATCTCCATCGCTCAGTACCTGGTGAGCGACCGTCCCATCATGCGTTACAGCGTCGAGCGTGGTTCCGACAAG
ATCTGCCGTCACAAGCTGTTCCAGGAGATGTCCTCCTTCGAGCAGATGCGCCTGTTCAAGACTCTGAGCTCCATCAGC
CTGTCCCTGATCAACTCCATGAAGACTAGCTTCTCCTCCCGTCTGCTGGTGAACGAGCGCGAATTTTCTAAGTACTTC
GGCAACGTGCGTCTGCGTGAGTGCTACGCTCAGCGCTTCTACCTGGCTGAGAGCCTGGTCGGCTTCCTGTTCTACCAG
AAGACCGGCGAACGCTCCCGCTGCTACAGCGTCTACCTGTCCGACAATGGTGTGATGAGCGAGCAGGGTTCCTTCTAC
TGCGACCCTAAGCGCTTCTTCCTGCCTGTGTTCTCCGACGAAGTGCTGGCCGGTATGTGCGAAGAGATGACCAGCTGG
CTGGACTTCGACACCGGTCTGATGAACGACACTGGTCCTATCCTGCGCCTGCTGGTGCTGGCCATCCTGTGCTCCCCT
AGCAAGCGTAACCAGACCTTCCTGCAGGGTCTGCGTTACTTCCTGATGGCTTTCGCTAACCAGATCCACCACATCGAC
CTGATCTCCAAGCTGGTCGTGGAATGCAAGTCCAGCAGCGAGGTCGTGGTCCAGCGCCTGGCTGTGGGTCTGTTCATC
CGTCTGCTGGGCGGTGAGAGCGACGCCAGCAGCTTCTTCTCCCGTCGTTTCAAGTACCTGCTGAACGTCAGCTACCTG
TGCCACCTGATCACCAAGGAGACCCCTGACCGTCTGACTGACCAGATCAAGTGCTTCGAAAAGTTCATCGAACCCAAG
GTCAAGTTCGGTTGCGCTGTCGTCAACCCTTCCCTGAACGGCAAGCTGACTGTCGACCAGGAAGACATCATGATCAAC
GGTCTGAAGAAGTTCTTCAGCAAGAGCCTGCGTGACACCGAGGACGTCCAGACTCCTGGCGTGTGCAAGGAACTGCTG
AACTACTGCGTCTCCCTGTTCAACCGCGGCAAGCTGAAGGTGTCCGGTGAGCTGAAGAACAACCCCTTCCGCCCCAAC
ATCACTAGCACCGCCCTGGACCTGAGCAGCAACAAGAGCGTCGTCATCCCCAAGCTGGACGAGCTGGGTAACATCCTG
AGCACTTACGACAAGGAAAAGCTGGTGAGCGCCTGCGTGTCCAGCATGGCCGAACGCTTCAAGACTAAGGGTCGTTAC
AACCTGGACCCCGAAAGCACCGACTACCTGATCCTGAAGAACCTGACTGGTCTGGTCTCCGCTGGCCCCAAGGCTAAG
TCCTCCCAGGAGGAACTGAGCCTGATGTACGAGACTCTGACTGAGGAGCAGGTGGAGAGCTTCAACGAGATCAAGTAC
GACGTGCAGGTCGCTCTGGCTAAGATGGCTGACAACTCCGTCAACACCCGTATCAAGAACCTGGGTCGCGCCGACAAC
TCCGTGAAGAACGGCAACAACCCCCTGGACAACCTGTGGAGCCCCTTCGGCGTGATGAAGGAAATCCGTGCCGAGGTG
AGCCTGCACGAAGTCAAGGACTTCGACCCCGACGTCCTGCCTAGCGACGTCTACAAGGAACTGTGCGACGCTGTGTAC
AAGAGCAGCGAGAAGTGCAACTTCTTCCTGGAGGAAGTGCTGGACGTCTGCCCCCTGGGTCTGCTGCTGAAGAACCTC
ACTACCTCCAGCTACATGGAAGAAGAGTACTTCATGTGCTTCAAGTACCTCCTGATCCAGGGCCACTTCGACCAGAAG
CTGGGTTCCTACGAACACAAGAGCCGCTCCCGTCTGGGCTTCACTGACGAGACTCTGCGTCTGAAGGACGAGGTCCGT
CTGAGCATCCGTGAGTCCAACAGCGAAGCTATCGCCGACAAGCTGGACAAGTCCTACTTCACCAACGCTGCCCTGCGT
AACCTGTGCTTCTACTCCGAAGACAGCCCCACTGAGTTCACCAGCATCAGCTCCAACAGCGGTAACCTGAAGTTCGGT
CTGTCCTACAAGGAGCAGGTGGGCTCCAACCGCGAGCTGTACGTGGGTGACCTGAACACCAAGCTGATGACCCGCCTG
GTGGAGGACTTCAGCGAGGCCGTGGGTAACTCCATGAAATACACTTGCCTGAACTCCGAAAAGGAGTTCGAGCGTGCT
ATCTGCGACATGAAGATGGCCGTCAACAACGGTGACCTGTCCTGCAGCTACGACCACTCCAAGTGGGGTCCCACCATG
TCCCCTGCCCTGTTCCTGGCTCTGCTGCAGATGCTGGAGCTGCGTACTCCTGTGGACCGCAGCAAGATCGACCTGGAC
TCCGTCAAGTCCATCCTGAAGTGGCACCTGCACAAGGTGGTGGAGGTCCCCATCAACGTCGCCGAAGCCTACTGCATC
GGTAAACTGAAGCGCAGCCTGGGCCTCATGGGCTGCGGCTCTACTAGCCTGAGCGAGGAATTTTTCCACCAGACTATG
CAGCTGTCCGGCCAGATCCCCTCCCACATCATGTCCGTCCTGGACATGGGTCAGGGTATCCTGCACAACACCTCCGAC
CTGTACGGCCTGATCACCGAGCAGTTCCTGTGCTACGCCCTGGACCTCCTGTACGACGTGATCCCCGTGAGCTACACC
TCCAGCGACGACCAGATCACCCTGGTGAAGACTCCCTCCCTGGACATCGAAGGTGGTTCCGACGCCGCCGAGTGGCTG
GAGATGATCTGCTTCCACGAGTTCCTGTCCAGCAAGCTGAACAAGTTCGTCTCCCCCAAGAGCGTGATCGGTACTTTC
GTCGCTGAGTTTAAAAGCCGCTTCTTCGTGATGGGCGAAGAAACCCCTCTGCTGACTAAGTTCGTGTCCGCCGCTCTG
CACAACGTGAAGTGCAAGACTCCCACTCAGCTGTCCGAAACTATCGACACCATCTGCGACCAGTGCATCGCCAACGGC
GTGTCCACTAAGATCGTGGCTCGTATCTCCAAGCGCGTGAACCAGCTGATCCGCTACAGCGGTTACGGCGACACTCCT
TTCGGCGCTATCGAAGACCAGGACGTGAAGGACTGGGTGGACGGCAGCCGTGGCTACCGTCTGCAGCGCAAGATCGAG
GCTATCTTCTACGACGACAAGGAGACTTCCTTCATCCGTAACTGCGCCCGTAAGGTCTTCAATGACATCAAGCGCGGC
CGCATCTTCGAAGAGAACCTGATCAACCTGATCGGCCGTGGTGGTGACGAGGCCCTGACCGGTTTCCTGCAGTACGCC
```

```
GGTTGCAGCGAACAGGAGGTCAACCGCGTGCTGAACTACCGCTGGGTGAACCTGAGCAGCTTCGGTGACCTGCGCCTG
GTCCTGCGCACCAAGCTGATGACTTCCCGCCGTGTGCTGGAGCGTGAGGAAGTGCCCACTCTGATCAAGACCCTGCAG
AGCAAGCTGTCCCGCAACTTCACCAAGGGCGTGAAGAAGATCCTGGCTGAGTCTATCAACAAGTCCGCCTTCCAGTCC
AGCGTGGCTTCCGGTTTCATCGGTTTCTGCAAGAGCATGGGTTCCAAGTGCGTGCGTGACGGTAAAGGTGGCTTCCTG
TACATCAAGGAGGTGTACTCCGGTATCAACGTCTGCATCTGCGAAATCTGCGCCCTGAAGCCTAAGATCATCTACTGC
AACGACTCCCTGAACAAGGTCAGCCAGTTCAGCAAGCCTATCCTGTGGGACTACTTCTCCCTGGTGCTGACCAACGCT
TGCGAGCTGGGCGAGTGGGTCTTCTCCACCGTGAAGGAGCCTCAGAAGCCTCTGGTGCTGAACAACCAGAACTTCTTC
TGGGCTGTGAAGCCTAAGGTGGTGCGTCAGATCGAAGACCAACTGGGCATGAACCACGTGCTGCAGTCCATCCGCCGC
AACTACCCTGTGCTGTTCGACGAACACCTGGCTCCCTTCATGAACGACCTGCAGGTCAGCCGTACTATGGATAGCGGC
CGTCTGAAGTTCCTGGACGTGTGCATCGCTCTGGACATGATGAACGAAAACCTGGGTATCATCAGCCACCTGCTGAAG
ACTCGTGACAACTCCGTTTACATCGTGAAGCAGTCCGACTGCGCTCTGGCCCACATCCGCCAGTCCTCCTACACTGAC
TGGGAGCTGGGTCTGAGCCCTCAGCAGATCTGCACCAACTTCAAGACCCAGCTGGTGCTGAGCAGCATGGTGAACCCC
CTGGTGCTGTCTACTTCCTGCCTGAAGAGCTTCTTCTGGTTCAACGAAGTCCTGGAACTGGAGGACGACTCCCAGATC
GAACTGGCCGAACTGACTGACTTCGCTCTGATGGTCAAGAACCAGAACGTCTCCCGTGCCATGTTCGTCGAGGACATC
GCTATGGGTTACGTCGTGAGCAACTTCGAAGGTGTGCGCATCTCCCTGAGCAACGTGATGGTGGACGGTGTCCAGCTG
CCCCCCAAGGAAAAGGCTCCTGACGTGGGCGTCCTGTTCGGTCTGAAGGCTGAAAACGTGATCGTGGGCCTGGTCGTC
CAGATCGACCACGTCCGCATGAGCACTAAGTTCAAGCTCCGCCGTAAGATGGTCTACAGCTTCAGCCTGGAGTGCACT
ATGGACGTCGGTGACATCCAGAACAAGGAGGTCATTCTGAAGGTGGTGGCCGTCGACCAGTCCGTCAGCGGTTCCGGT
GGCAACCACATGCTGCTGGACGGTGTGCCTGTCATCGCTTCCCTGCCTCTGTTCACCGGCCAGGCTAGCTTCGACCTG
GCCGCTATGCTGATCGAGAGCAACCTGGCCGGCAGCAACGACAACTTCCTGATGAGCAACGTCACTCTGGACCTGGGC
GGCTTCTCCCCCGAGCTGAGCGACAAGTACTCCTACCGTCTGTCCGGCCCCGAAAACCAGGAAGACCCTCTGGTCCTG
AAGGACGGCGCCTTCTACGTGGGTGGCGAACGTCTGTCCACCTACAAGGTCGAGCTGACCGGTGACCTGGTGGTCAAG
GCCCTGGGTGCCCTGGAGGACGATGAGGGCGTCGTGAGCATGCTGCACCAGCTGTGGCCTTACCTGAAGGCCACTAGC
CAGGTCATTCTGTTCCAGCAGGAGGACTTCACCATCGTCCACGACCTGTACAAGATCCAGCTGACCAAGTCCATCGAG
TCCTTCGGTGAGTGGATCGAGTTCACTAACTTCAAGGTGGCTTACTCCAAGTCCCTGAAGGAGCTGGTCATCTCCGAC
ACTCAGGGTAGCTTCCGCCTGAAGGGCGTCATGTGCCGCCCCCTGGCTAACACTCTGCAGGTGGAAGACATCGAGTGG
TCCCACCCTCAGTTCGAAAAGGGCGGCGGTAGCGGCGGTGGCTCCGGTGGTTCAAGCGCTTGGAGCCACCCCCAGTTC
GAAAAATAA
```

b The codon-optimized DNA sequence of MACV Z gene

```
ATGGGCAACTGCAACAAGCCCCCCAAGCGCCCTCCCAACACCCAGACTTCCGCTGCCCAGCCTTCCGCCGAGTTCCGT
CGTACCGCTCTGCCCTCCCTGTACGGCCGTTACAACTGCAAGTGCTGCTGGTTCGCTGACACCAACCTGATCACTTGC
AACGACCACTACCTGTGCCTGCGCTGCCACCAGACTATGCTGCGTAACAGCGAACTGTGCCACATCTGCTGGAAGCCC
CTGCCCACTTCCATCACTGTCCCCGTGGAGCCCTCCGCTCCTCCCCCTTAA
```

c The codon-optimized DNA sequence of LASV Z gene

```
ATGGGCAATAAACAGGCCAAAGCACCGGAAAGTAAAGATAGCCCGCGTGCCAGCCTGATTCCGGATGCCACCCATCTG
GGCCCGCAGTTTTGCAAAAGTTGTTGGTTTGAAAATAAGGGTCTGGTTGAATGTAATAATCATTATCTGTGTCTGAAC
TGCCTGACCCTGCTGCTGAGCGTTAGTAATCGCTGCCCGATTTGTAAAATGCCGCTGCCGACCAAACTGCGTCCGAGC
GCCGCCCCTACCGCACCTCCTACAGGTGCCGCCGATAGCATTCGCCCGCCGCCTTATAGTCCGTAA
```

d The codon-optimized DNA sequence of LCMV Z gene

```
ATGGGTCAGGGTAAAAGTCGTGAAGAAAAAGGTACAAATAGTACCAATCGTGCCGAAATTCTGCCGGATACCACCTAT
CTGGGCCCGCTGAGCTGTAAAAGCTGCTGGCAGAAATTTGATAGTCTGGTGCGTTGCCATGATCATTATCTGTGTCGC
CATTGTCTGAATCTGCTGCTGAGCGTTAGCGATCGTTGCCCGCTGTGTAAATATCCGCTGCCGACCCGCCTGAAAATT
AGCACCGCCCCGAGTAGTCCGCCGCCGTATGAAGAATAA
```

e The codon-optimized DNA sequence of JUNV Z gene

```
ATGGGCAATTGTAATGGTGCAAGTAAAAGCAATCAGCCGGATAGCAGCCGCGTTACCCAGCCGGCCGCCGAATTTCGT
CGCGTTGCACATAGCAGTCTGTATGGTCGCTATAATTGTAAATGTTGCTGGTTTGCCGATACCAATCTGATTACCTGT
AATGATCATTATCTGTGTCTGCGTTGTCATCAGGTTATGCTGCGTAATAGCGATCTGTGCAATATTTGCTGGAAACCG
CTGCCGACCACCATTACCGTGCCGGTGGAACCGACCGCCCCGCCTCCTTAA
```

f The codon-optimized DNA sequence of SABV Z gene

```
ATGGGTAATAGCAAAAGCAAAAGTAAGCTGAGCGCAAATCAGTATGAACAGCAGACCGTTAATAGTACCAAACAGGTG
GCAATTCTGAAACGCCAGGCAGAACCGAGCCTGTATGGTCGCCATAATTGTCGTTGTTGCTGGTTTGCAAATACCAAT
CTGATTAAGTGTAGTGATCATTATATCTGTCTGAAATGCCTGAATATTATGCTGGGTAAAAGCAGCTTTTGTGATATT
TGCGGCGAAGAACTGCCGACCAGTATTGTTGTTCCGATTGAACCGAGTGCCCCGCCGCCGGAAGATTAA
```

g The codon-optimized DNA sequence of CHAV Z gene

```
ATGGGCAATACCAAAACCAAAGATCGCCAGTATCAGAGCAATAGTAGCCAGCCGACCAATACCAGCGCCCCGGTTCTG
CTGCGTCGCCAGGCTGAACCGAGCCTGTATGGTCGTCATAATTGCCGTTGTTGCTGGTTTGCAGATACCAATCTGGTT
AATTGTAGTAATCATTACCTGTGTCTGAAATGTCTGAATACCATGCTGCGTCGCAGTAATCTGTGTGATATTTGCGGC
GAAGAACTGCCGACCACCATTATTGTGCCGGTTGAACCGAGTGCCCCGCTGCCGGGTCAGTAA
```

h The codon-optimized DNA sequence of TCRV Z gene

```
ATGGGTAATTGTAACCGTACCCAGAAACCGAGCAGCAGCAGCAATAATCTGGAAAAACCGCCGCAGGCCGCCGAATTT
CGCCGCACAGCCGAACCGAGCCTGTATGGTCGTTATAATTGTAAATGCTGTTGGTTTGCAGATAAAAATCTGATTACC
TGTAGTGATCATTACCTGTGTCTGCGCTGCCATCAGATTATGCTGCGCAATAGTGAACTGTGCAATATTTGTTGGAAA
CCGCTGCCGACCAGTATTCGTGTTCCGCTGGAAGCAAGTGCCCCGGATCTGTAA
```

Supplementary Table 2. The codon-optimized DNA sequences of L and Z genes. MACV L (**a**); MACV Z (**b**); LASV Z (**c**); LCMV Z (**d**); JUNV Z (**e**); SABV Z (**f**); CHAV Z (**g**); TCRV Z (**h**).

Primers	Sequence
3′ vRNA	5′-GCGUGUCACCUAGGAUCCG-3′
5′ vRNA	5′-CGCACCGGGGAUCCUAGGC-3′

Supplementary Table 3. The vRNA primers for *in vitro* polymerase activity assays.
