# Supplementary material for: Structure of Machupo virus polymerase in complex with matrix protein Z
Source: Nat Commun. 2021 Oct 25;12:6163. doi: 10.1038/s41467-021-26432-3 (PMC8546121; doi:10.1038/s41467-021-26432-3)
Supplement: Supplementary file 3 — Reporting Summary [file 41467_2021_26432_MOESM3_ESM.pdf]

nature portfolio

Corresponding author(s): Xinzheng Zhang

Last updated by author(s): 2021-09-22

Reporting Summary

Nature Portfolio wishes to improve the reproducibility of the work that we publish. This form provides structure for consistency and transparency in reporting. For further information on Nature Portfolio policies, see our Editorial Policies and the Editorial Policy Checklist.

Statistics

For all statistical analyses, confirm that the following items are present in the figure legend, table legend, main text, or Methods section.

n/a	Confirmed	
☐	☒	The exact sample size (*n*) for each experimental group/condition, given as a discrete number and unit of measurement
☐	☒	A statement on whether measurements were taken from distinct samples or whether the same sample was measured repeatedly
☒	☐	The statistical test(s) used AND whether they are one- or two-sided<br>*Only common tests should be described solely by name; describe more complex techniques in the Methods section.*
☒	☐	A description of all covariates tested
☒	☐	A description of any assumptions or corrections, such as tests of normality and adjustment for multiple comparisons
☒	☐	A full description of the statistical parameters including central tendency (e.g. means) or other basic estimates (e.g. regression coefficient) AND variation (e.g. standard deviation) or associated estimates of uncertainty (e.g. confidence intervals)
☒	☐	For null hypothesis testing, the test statistic (e.g. F, t, r) with confidence intervals, effect sizes, degrees of freedom and P value noted<br>*Give P values as exact values whenever suitable.*
☒	☐	For Bayesian analysis, information on the choice of priors and Markov chain Monte Carlo settings
☒	☐	For hierarchical and complex designs, identification of the appropriate level for tests and full reporting of outcomes
☒	☐	Estimates of effect sizes (e.g. Cohen's *d*, Pearson's *r*), indicating how they were calculated

Our web collection on statistics for biologists contains articles on many of the points above.

Software and code

Policy information about availability of computer code

Data collection	SerialEM-3.7 for cryo-EM data collection
Data analysis	MotionCor2-1.1.0, CTFFIND 4.1, Gautomatch-v0.56, RELION-3.08, ResMap-1.1.4, UCSF Chimera-1.12, Coot-0.8.8, PHENIX-1.13, PyMOL-2.0.8, Phyre2

For manuscripts utilizing custom algorithms or software that are central to the research but not yet described in published literature, software must be made available to editors and reviewers. We strongly encourage code deposition in a community repository (e.g. GitHub). See the Nature Portfolio guidelines for submitting code & software for further information.

Data

Policy information about availability of data

All manuscripts must include a data availability statement. This statement should provide the following information, where applicable:

- Accession codes, unique identifiers, or web links for publicly available datasets
- A description of any restrictions on data availability
- For clinical datasets or third party data, please ensure that the statement adheres to our policy

The cryo-EM density maps and corresponding coordinates have been deposited in the Electron Microscopy Data Bank (EMDB) and Protein Data Bank (PDB), respectively, under the accession codes: monomeric L-Z complex (EMD-31975 [https://www.emdataresource.org/EMD-31975], 7VGQ [http://doi.org/10.2210/pdb7vgq/pdb]), dimeric L-Z complex (EMD-31983 [https://www.emdataresource.org/EMD-31983], 7VH1 [http://doi.org/10.2210/pdb7vh1/pdb]), monomeric apo L (EMD-31985 [https://www.emdataresource.org/EMD-31985], 7VH3 [http://doi.org/10.2210/pdb7vh3/pdb]) and dimeric apo L (EMD-31984 [https://www.emdataresource.org/EMD-31984], 7VH2 [http://doi.org/10.2210/pdb7vh2/pdb]). The source data for Fig 1c, Supplementary Figure 1b & c, Supplementary Figure 6a, Supplementary Figure 7c & d are provided with this paper. All data that support the findings of this study are available from the corresponding authors on [illegible]

reasonable request.
For single particle cryo-EM reconstructions, the density maps of MACV L monomer (EMD-0707) and dimer (EMD-0710) were used as the initial models for the monomeric and dimeric MACV L-Z complex, respectively. The coordinates of MACV L (6KLD) and LASV Z (5I72) were used for model building of the monomeric MACV L-Z complex. The coordinates of MACV L monomer (6KLD), vRNA-bound dimer (6KLH) and L-Z complex (7CKM), JUNV L-Z complex (7EJU), LASV L (6KLC) and Z (2M1S and 5I72) were used for structural analysis.
The protein sequences of Z: MACV, Q6IUF9; JUNV, Q6IVU5; GTOV, Q6UY71; TCRV, Q88470, SABV, Q6UY62, CHAV, B2C4J2; LATV, A9JR44; OLVV, B0BLK7; WWAV, B2ZDY1; PICV, Q915A4; PIRV, Q6RSS3; LASV, O73557; MOPV, G3LUW9; MOBV, Q27YE6; IPPV, Q27YE2; LCMV, P18541; LUJV, C5ILC3; and L: MACV, Q6IUF8; JUNV, Q6XQI4; GTOV, Q6UY70; TCRV, P20430; SABV, Q6UY61; CHAV, B2C4J3; OLVV, Q6XQH7; PIRV, Q6RSS2; IPPV, Q27YE1; LASV, O09705; MOBV, Q27YE5; LCMV, P14240 are used for sequence analysis.

Field-specific reporting

Please select the one below that is the best fit for your research. If you are not sure, read the appropriate sections before making your selection.

☒ Life sciences ☐ Behavioural & social sciences ☐ Ecological, evolutionary & environmental sciences

For a reference copy of the document with all sections, see nature.com/documents/nr-reporting-summary-flat.pdf

Life sciences study design

All studies must disclose on these points even when the disclosure is negative.

Sample size	No statistical methods were used to predetermine sample size. For cryo-EM study, data were collected for at least one day until the density maps could not be improved. For all the functional experiments, the sample size was determined according to the reproducibility of the experiments.
Data exclusions	No data has been excluded.
Replication	The protein expression and purification, pull-down assay and polymerase activity assay reported were done more than two independent times with consistent results.
Randomization	Randomization is not relevant to this study, because no grouping was needed for the assays.
Blinding	Blinding is not applied to this study as the experiments are performed under defined conditions.

Reporting for specific materials, systems and methods

We require information from authors about some types of materials, experimental systems and methods used in many studies. Here, indicate whether each material, system or method listed is relevant to your study. If you are not sure if a list item applies to your research, read the appropriate section before selecting a response.

Materials & experimental systems

n/a	Involved in the study	
☒	☐	Antibodies
☐	☒	Eukaryotic cell lines
☒	☐	Palaeontology and archaeology
☒	☐	Animals and other organisms
☒	☐	Human research participants
☒	☐	Clinical data
☒	☐	Dual use research of concern

Methods

n/a	Involved in the study	
☒	☐	ChIP-seq
☒	☐	Flow cytometry
☒	☐	MRI-based neuroimaging

Eukaryotic cell lines

Policy information about cell lines

Cell line source(s)	Insect cells Hi5 were purchased from Thermo Fisher Scientific.
Authentication	Hi5 cells were purchased and routinely maintained in the lab, not authenticated experimentally for this study.
Mycoplasma contamination	Hi5 cells were tested negative for mycoplasma contamination.
Commonly misidentified lines (See ICLAC register)	No commonly misidentified cell lines were using in this study.
